# Dipstick Leukocyturia as a Kidney Damage Biomarker in Rural Uganda and Kenya

**DOI:** 10.1016/j.xkme.2024.100895

**Published:** 2024-08-14

**Authors:** Sahra Mohamed, Chi-Yuan Hsu, Edwin D. Charlebois, Jane Kabami, Mucunguzi Atukunda, James Ayieko, Gordon Orori, Matthew D. Hickey, Maya Petersen, Moses R. Kamya, Diane Havlir, Michelle M. Estrella, Anthony N. Muiru

**Affiliations:** 1 Institute for Global Health Sciences, University of California-San Francisco, San Francisco, CA; 2 Department of Medicine, Division of Nephrology, University of California, San Francisco, School of Medicine, San Francisco, CA; 3 Department of Medicine, Center for AIDS Prevention Studies, Division of Prevention Science, University of California, San Francisco, San Francisco, CA; 4 Infectious Diseases Research Collaboration, Kampala, Uganda; 5 Kenya Medical Research Institute, Nairobi, Kenya; 6 Department of Medicine, Division of HIV, ID and Global Medicine, University of California, San Francisco, CA; 7 Division of Epidemiology and Biostatistics, School of Public Health, University of California, Berkeley, Berkeley, CA; 8 Department of Medicine, Makerere University, Kampala, Uganda

To the Editor:

The presence of sterile leukocyturia may provide diagnostic and prognostic insights into kidney health. For instance, leukocyturia among patients with chronic kidney disease (CKD) of unknown etiology is associated with lower estimated glomerular filtration rate, interstitial nephritis, and tubular atrophy on kidney biopsies.^1^ We previously reported a high prevalence of leukocyturia in sub-Saharan Africa.^2^ We now seek to better understand the risk factors associated with leukocyturia.

To estimate kidney disease prevalence in rural East Africa from 2016-2017, we measured serum creatinine and performed urine dipstick urinalysis.^2^ We interpreted dipstick urinalysis showing leukocyte esterase (≥1+) with nitrate negative results, as leukocyturia not explained by urinary tract infection. We included 3,462 community representative participants from Eastern Uganda, Southwestern Uganda, and Western Kenya.^2^ A detailed description of the study population and our sampling design can be found in the supplemental section (Item S1).

We applied weighted multivariable log-link Poisson regression models with robust standard errors to estimate the adjusted population-level prevalence ratios for leukocyturia and explored associations with risk factors including geographic region, serum creatinine, dipstick proteinuria, sociodemographic factors, smoking, alcohol, diabetes, hypertension, HIV, use of nonsteroidal anti-inflammatory drugs, and traditional herbal medicines. These factors were chosen based on their previous associations with kidney disease in sub-Saharan Africa and other regions.^3^, ^4^, ^5^, ^6^ We hypothesized that leukocyturia may indicate kidney damage.

We also evaluated whether certain environmental conditions, such as the mean surface air temperatures and high altitudes, were associated with leukocyturia and whether leukocyturia is associated with prevalent CKD (defined as an estimated glomerular filtration rate of <60 mL/min/1.73m^2^ or dipstick proteinuria ≥1+).

The baseline characteristics of the sampled participants and the weighted population-level estimates are shown in Table 1 and Table S1 respectively.

**Table 1:** Baseline Characteristics of the Sampled Participants

	No Leukocyturia n = 3,163	Leukocyturia n = 299	Total N = 3,462
Region			
Eastern Uganda	987 (31.2)	164 (54.8)	1,151 (33.2)
Southwestern Uganda	732 (23.1)	98 (32.8)	830 (24.0)
Western Kenya	1,444 (45.7)	37 (12.4)	1,481 (42.8)
Sex			
Female	2,025 (64.0)	228 (76.3)	2,253 (65.1)
Male	1,137 (36.0)	71 (23.7)	1,208 (34.9)
Age categories			
18-29 y	613 (19.4)	58 (19.4)	671 (19.4)
30-44 y	1,270 (40.1)	97 (32.4)	1,367 (39.5)
45-59 y	800 (25.3)	89 (29.8)	889 (25.7)
≥60 y	480 (15.2)	55 (18.4)	535 (15.4)
Education level			
No formal education	459 (14.8)	82 (27.7)	541 (15.9)
Primary school	2,174 (70.0)	172 (58.1)	2,346 (68.9)
Secondary school and beyond	474 (15.2)	42 (14.2)	516 (15.2)
Wealth index/score^a^			
1st quintile	587 (18.8)	71 (24.0)	658 (19.3)
2nd quintile	517 (16.6)	56 (18.9)	573 (16.8)
3rd quintile	630 (20.2)	53 (17.9)	683 (20.0)
4th quintile	685 (22.0)	67 (22.6)	752 (22.0)
5th quintile	698 (22.4)	49 (16.6)	747 (21.9)
Farmer	1,996 (64.2)	231 (78.0)	2,227 (65.4)
Smoking status			
Never smoker	2,714 (87.3)	260 (87.5)	2,974 (87.3)
Current	192 (6.2)	17 (5.7)	209 (6.1)
Past	203 (6.5)	20 (6.7)	223 (6.6)
Any current alcohol use	291 (10.1)	34 (12.4)	325 (10.3)
CKD^b^	240 (7.6)	79 (26.7)	319 (9.2)
HIV-positive	1,378 (44.2)	143 (48.3)	1,521 (44.6)
Diabetes mellitus	121 (3.9)	13 (4.4)	134 (3.9)
Hypertension	596 (19.3)	56 (18.9)	652 (19.2)
Any NSAID use over the previous 90 days	1,594 (51.3)	128 (43.13)	1,722 (50.5)
Any traditional medicine use over the previous 90 days	833 (26.8)	89 (30.0)	922 (27.1)

Abbreviations: NSAID, nonsteroidal anti-inflammatory drugs; CKD, chronic kidney disease.

a Wealth index/score (divided in quintiles) was calculated using principal components analysis based on ownership of livestock and other household items.

b Defined as serum creatinine estimated glomerular filtration rate of <60 mL/min/1.73m^2^ or proteinuria (urine dipstick ≥1+).

We observed a striking geographic variation in the prevalence of leukocyturia, with notably higher rates in Uganda (11.2% in Eastern and 8.7% in Southwestern Uganda) compared with 1.6% in Kenya.

In our fully adjusted model, residences in Eastern and Southwestern Uganda (compared with Kenya) were strongly associated with leukocyturia (adjusted prevalence ratio [aPR], 7.51; 95% confidence interval [CI], 4.36-12.95 and aPR, 7.78; 95% CI, 4.48-13.51). In addition, female sex (aPR, 1.80; 95% CI, 1.14-2.84), primary school education (aPR, 2.07; 95% CI, 1.04-4.12, compared with secondary school and beyond), and dipstick proteinuria (aPR, 3.58; 95% CI, 2.35-5.45) were associated with leukocyturia. We did not observe any significant associations between HIV, diabetes, or hypertension with leukocyturia (Table 2).

**Table 2:** Unadjusted and Adjusted Association of Potential Risk Factors With Leukocyturia

	Leukocyturia			
	Unadjusted Prevalence Ratio (95% CI)	*P* Value	Adjusted Prevalence Ratio (95% CI)	*P* Value
Region				
Eastern Uganda	7.20 (4.51-11.51)	<0.001	7.51 (4.36-12.95)^a^	<0.001^a^
Southwestern Uganda	5.58 (3.34-9.33)	<0.001	7.78 (4.48-13.51)^a^	<0.001^a^
Western Kenya	Reference		Reference	
Female	2.22 (1.40-3.52)	0.001	1.80 (1.14-2.84)^a^	0.01^a^
Age				
18-29 y	Reference		Reference	
30-44 y	0.68 (0.42-1.10)	0.11	0.70 (0.44-1.12)	0.14
45-59 y	1.04 (0.65-1.68)	0.86	1.00 (0.63-1.59)	0.99
≥60 y	1.39 (0.86-2.24)	0.18	1.43 (0.88-2.33)	0.15
Education level				
No formal education	3.27 (1.62-6.60)	0.001	1.95 (0.95-3.99)	0.07
Primary school	1.73 (0.87-3.42)	0.12	2.07 (1.04-4.12)^a^	0.04^a^
Secondary school and beyond	Reference		Reference	
Wealth index				
1st quintile (least wealth)	Reference		Reference	
2nd quintile (less wealth)	1.04 (0.62-1.72)	0.89	1.02 (0.63-1.67)	0.91
3rd quintile (middle wealth)	0.74 (0.41-1.32)	0.30	0.98 (0.54-1.78)	0.95
4th quintile (more wealth)	0.96 (0.57-1.61)	0.87	1.24 (0.76-2.03)	0.39
5th quintile (most wealth)	0.73 (0.41-1.32)	0.30	1.05 (0.61-1.81)	0.85
Farmer	1.93 (1.19-3.13)	0.01	1.04 (0.67-1.60)	0.86
Smoking status				
Current smoker	0.89 (0.47-1.69)	0.73	0.95 (0.48-1.88)	0.88
Past smoker	0.88 (0.47-1.64)	0.69	0.80 (0.43-1.49)	0.48
Any alcohol use	0.68 (0.37-1.26)	0.22	0.62 (0.32-1.20)	0.16
Dipstick proteinuria	5.94 (4.08-8.65)	<0.001	3.58 (2.35-5.45)^a^	<0.001^a^
Serum creatinine	0.32 (0.13-0.77)	0.01	1.26 (0.78-2.06)	0.35
HIV-positive	0.73 (0.55-0.98)	0.04	1.14 (0.82-1.59)	0.43
Diabetes mellitus	0.94 (0.44-1.99)	0.87	0.69 (0.31-1.52)	0.36
Hypertension	0.97 (0.64-1.48)	0.90	0.70 (0.46-1.07)	0.10
Any NSAIDs use over the previous 90 days	0.75 (0.52-1.09)	0.13	1.12 (0.78-1.60)	0.55
Any traditional medicine use over the previous 90 days	1.11 (0.77-1.60)	0.57	0.86 (0.61-1.24)	0.43

*Note:* Adjusted model included geographic region, serum creatinine, dipstick proteinuria, sociodemographic factors, health habits (smoking and alcohol), diabetes, hypertension, HIV, proteinuria, hematuria, use of NSAIDs, and traditional herbal medicines.

Abbreviations: CI, confidence interval; NSAID, nonsteroidal anti-inflammatory drugs.

a Significant values.

We found that the mean surface air temperatures in 2016 were 23.7 °C, 20.2 °C, and 22.1 °C, and the mean altitudes were 1,131 meters, 1,470 meters, and 1,397 meters in Eastern Uganda, Southwestern Uganda, and Kenya, respectively.^7^^,^^8^ However, neither the mean surface air temperatures nor altitudes were associated with leukocyturia (Table S2).

Leukocyturia was associated with CKD in the unadjusted (prevalence ratio, 4.81; 95% CI, 3.27-7.09) and fully adjusted models (aPR, 3.54; 95% CI, 2.20-5.68).

Given the patterns of leukocyturia in our study—highly variable geographic distribution between study communities and association with CKD—we postulate that leukocyturia may represent a marker of kidney damage in this region.

The observed geographical variations in leukocyturia distribution may suggest that these differences are attributed to environmental exposures. However, we could not verify this as neither mean surface air temperatures nor altitudes were found to be associated with leukocyturia.

Differences in leukocyturia prevalence have been noted among farm workers in various job categories, hinting at a potential influence from farming-related environmental factors.^1^ Despite a larger proportion of Eastern Uganda and Southwestern Uganda participants identifying as farmers compared with those in Western Kenya, farming occupation was not associated with leukocyturia in our study. Further investigations are needed to elucidate the role of environmental exposures in leukocyturia in this region.

Population-based studies on asymptomatic leukocyturia are scarce,^9^ and our report contributes to the literature by delineating patterns and risk factors for leukocyturia in rural East Africa. However, we acknowledge several limitations. We did not do urine cultures to rule out bacterial infection. We were unable to establish the relationship between leukocyturia and longitudinal loss of kidney function. Additionally, we did not conduct microscopic examinations of urine sediment, and relying solely on dipstick urinalysis limits our ability to make definitive links between our findings and clinically relevant kidney disease.

The absence of robust health care and research infrastructure in the region^10^ prevents us from making further connections between leukocyturia and advanced CKD (for example, kidney failure treated with dialysis). We lacked information on endemic infections such as tuberculosis, which might have provided additional insights into the etiology of leukocyturia. Nevertheless, the presence of leukocyturia in the context of infection can indicate genitourinary involvement and potentially contribute to subsequent kidney disease.

In conclusion, we observed that sterile leukocyturia has a striking geographic variation—being highly prevalent in rural Uganda but not in Kenya and is associated with CKD. Leukocyturia may represent a valuable marker of kidney damage in this region, and future studies should further characterize leukocyturia in these communities to determine its clinical significance.

## Supplementary Material

Supplementary File (PDF) Item S1; Table S1 and S2.
