# Supplementary material for: Dipstick Leukocyturia as a Kidney Damage Biomarker in Rural Uganda and Kenya
Source: Kidney Med. 2024 Aug 14;6(10):100895. doi: 10.1016/j.xkme.2024.100895 (PMC11407920; doi:10.1016/j.xkme.2024.100895)
Supplement: Supplementary File (PDF) — Item S1; Table S1 and S2. [file mmc1.pdf]

Item S1: Description of Study Population and Sampling Design

This cross-sectional study is embedded within a large HIV test and treat clinical trial conducted in Uganda and Kenya (Sustainable East Africa Research in Community Health [SEARCH, NCT01864603]).[1] SEARCH was a 32-community cluster-randomized controlled trial with 355,848 participants living in 10 communities in eastern Uganda, 10 in southwestern Uganda, and 12 in western Kenya.[1] As described elsewhere, 100 households with at least one HIV-positive adult and 100 households without any HIV-positive adults were randomly selected to participate in SEARCH sub-studies.[2] For this sub-study, we chose one HIV-positive and one HIV-negative adult from the respective households in 22 SEARCH communities.[3] Each study country's institutional review boards approved the study, and all participants consented to participate.

To estimate the population-level prevalence of dipstick leukocyturia and understand its associated risk factors, we used inverse probability weighting to account for the sampling of households within each community and to adjust for incomplete ascertainment of leukocyturia among selected participants.[3-5] The household selection probability was estimated using empirical proportions specific to each community, and the probability of outcome measurement among selected participants was estimated with logistic regression controlling for age, gender, HIV serostatus, relationship to head of household, and region.[3]

Using these weights, we applied multivariable log-link Poisson regression models with robust standard errors to estimate the adjusted prevalence ratios for leukocyturia and explored associations with risk factors including geographic region, serum creatinine (Scr), dipstick proteinuria, sociodemographic factors, smoking, alcohol, diabetes, hypertension, HIV, use of non-steroidal anti-inflammatory drugs, and traditional herbal medicines. These factors were chosen based on their previous associations with kidney disease in sub-Saharan Africa and other regions.[6-9] We hypothesized that leukocyturia may indicate kidney damage. All analyses were conducted in Stata version 15 (StataCorp, College Station, Texas, USA).

Table S1: Population characteristics of adults in rural East Africa based on weighted SEARCH-CKD participants

	No Leukocyturia	Leukocyturia	Total
Region			
Eastern Uganda, %	33.5	56.2	35.1
Southwestern Uganda, %	28.4	35.8	28.9
Western Kenya, %	38.2	8.0	36.1
Sex			
Female, %	51.0	71.0	52.4
Male, %	49.0	29.0	47.6
Age categories			
18–29 years, %	37.2	39.6	37.3
30–44 years, %	34.4	24.2	33.7
45–59 years, %	17.5	19.6	17.7
≥ 60 years, %	10.9	16.6	11.3
Education level			
No formal education, %	10.9	21.7	11.6
Primary school, %	67.6	66.5	67.6
Secondary school and beyond, %	21.5	11.8	20.8
Wealth Index/score⊥			
1st quintile, %	15.4	17.7	15.6
2nd quintile, %	17.3	20.8	17.6
3rd quintile, %	21.2	17.6	21.0
4th quintile, %	21.7	23.8	21.8
5th quintile, %	24.4	20.1	24.1
Farmer, %	58.8	74.3	59.9
Smoking status			
Never smoker, %	88.8	90.0	88.9
Current, %	5.6	5.0	5.6
Past, %	5.6	5.0	5.6
Any current alcohol use, %	11.6	8.0	11.3
CKD, %	5.4	26.0	6.8
HIV-positive, %	9.6	7.1	9.4
Diabetes mellitus, %	3.8	3.5	3.7
Hypertension, %	17.0	16.6	17.0
Any NSAID use over the previous 90 days, %	48.9	41.3	48.4
Any traditional medicine use over the previous 90 days, %	27.7	30.1	27.9

⊥Wealth index/score (divided in quintiles) was calculated using principal components analysis based on ownership of livestock and other household items.
NSAID: nonsteroidal anti-inflammatory drugs
CKD: Chronic kidney disease defined as serum creatinine estimated glomerular filtration rate <60 mL/min/1.73m^2 or proteinuria (urine dipstick ≥1+)

Table S2: Exploratory analysis: Unadjusted and adjusted association of environmental risk factors with leukocyturia

	Unadjusted Prevalence Ratio (95% CI)	P value	Adjusted Prevalence Ratio (95% CI)	P value
Mean air temperature	1.08 (0.97-1.20)	0.18	0.97 (0.77-1.22)	0.81
Altitude	1.00 (1.00-1.00)	0.002	1.00 (1.00-1.00))	0.44

References

1. Havlir DV, Balzer LB, Charlebois ED, et al. HIV Testing and Treatment with the Use of a Community Health Approach in Rural Africa. *N Engl J Med*. Jul 18 2019;381(3):219-229. doi:10.1056/NEJMoa1809866
2. Jakubowski A, Kabami J, Balzer LB, et al. Effect of universal HIV testing and treatment on socioeconomic wellbeing in rural Kenya and Uganda: a cluster-randomised controlled trial. *Lancet Glob Health*. Jan 2022;10(1):e96-e104. doi:10.1016/s2214-109x(21)00458-7
3. Muiru AN, Charlebois ED, Balzer LB, et al. The epidemiology of chronic kidney disease (CKD) in rural East Africa: A population-based study. *PLoS One*. 2020;15(3):e0229649. doi:10.1371/journal.pone.0229649
4. Horvitz DG, Thompson DJ. A Generalization of Sampling Without Replacement From a Finite Universe. *Journal of the American Statistical Association*. 1952;47(260):663-685. doi:10.1080/01621459.1952.10483446
5. Hernan MA, Robins JM. Estimating causal effects from epidemiological data. *J Epidemiol Community Health*. Jul 2006;60(7):578-86. doi:10.1136/jech.2004.029496
6. Kalyesubula R, Wearne N, Semitala FC, Bowa K. HIV-associated renal and genitourinary comorbidities in Africa. *J Acquir Immune Defic Syndr*. Sep 1 2014;67 Suppl 1:S68-78. doi:10.1097/QAI.0000000000000259
7. Sandler DP, Burr FR, Weinberg CR. Nonsteroidal anti-inflammatory drugs and the risk for chronic renal disease. *Annals of internal medicine*. Aug 1 1991;115(3):165-72.
8. Jha V. Herbal medicines and chronic kidney disease. *Nephrology (Carlton)*. Jun 2010;15 Suppl 2:10-7. doi:10.1111/j.1440-1797.2010.01305.x
9. Hsu CY, Iribarren C, McCulloch CE, Darbinian J, Go AS. Risk factors for end-stage renal disease: 25-year follow-up. *Arch Intern Med*. Feb 23 2009;169(4):342-50.
